# Auf dem Weg zur Telemedizin: Erkenntnisse aus 28 Jahren psychosomatischer Medizin

**DOI:** 10.1007/s00608-020-00846-6

**Published:** 2020-11-30

**Authors:** Peter J. Scheer

**Affiliations:** 1 NoTube gemeinnützige GmbH, Kerschhoferweg 14, 8010 Graz, Österreich; 2 Psychosoamtik & Psychotherpie, Abteilung für allgemeine Pädiatrie, Univ.-Klinik für Kinder- und Jugendheilkunde, Medizinische Universität und Landeskrankenhaus Graz, Graz, Österreich

**Keywords:** Psychosomatische Medizin, Familien, Psychosomatische Störungen, Telemedizin, Nutritional problems, Psychosomatic medicine, Families, Psychosomatic disorders, Telemedicine, Ernährungsprobleme

## Abstract

Dieser Beitrag umfasst die Abschiedsrede (2012) zum Ende einer 28-jährigen Tätigkeit als Leiter der Psychosomatik und Psychotherapie innerhalb der Abteilung für allgemeine Pädiatrie der Universitätsklinik für Kinder- und Jugendheilkunde im Landeskrankenhaus Graz. Dabei wird das Beziehungsdreieck zwischen Arzt, Eltern und Kind betrachtet: Die Aufgabe des Arztes ist es, aufmerksam die verbalen und non-verbalen Äußerungen der Kinder, der Eltern und letzten Endes auch seine eigenen wahrzunehmen. In einem Prozess des Handelns und Behandelns soll ärztliches Tun Teil eines gemeinsamen Erlebens werden. Dazu ist Feinfühligkeit ebenso nötig wie Selbstkritik und Achtung vor den Familien, die sich uns anvertrauen. Dies umzusetzen gelang uns auch bei Kleinkindern mit Essstörungen oder bei Kindern, die an Sondendependenz leiden. Die Spin-off Initiative NoTube GmbH für Kinder zwischen Null und 8 Jahren, die an Essstörungen leiden, wird als Fortsetzung der langjährigen Tätigkeit an der Medizinischen Universität Graz vorgestellt. Die Erfahrungen des psychosomatischen Verstehens und Handelns, die an der Klinik erworben wurden, können so – nach der Versetzung in den Ruhestand – weiterhin hunderten Familien zugutekommen. Durch die Errichtung eines Ambulatoriums und v.a. durch das Angebot, Familien telemedizinisch zu begleiten, ist es eine Medizin, die besonders in Zeiten der Covid-19-Pandemie den Weg in die Zukunft weist.

## Abstract (en)

This article is based on the script of my farewell-speech at the University Hospital for Children and Adolescents in 2012. I served for 28 years as Head of the Psychosomatic and Psychotherapeutic unit as part of the Division for General Pediatrics, which was imbedded in the Department for Pediatrics. In these years I learned to focus on the triangle between doctor, parents, and child: To observe and recognize the verbal and non-verbal expressions of the children, the parents, and also his own is one of the doctor’s key tasks. Within this process of acting and treating, practising medicine should become part of a commonly lived experience. This requires sensitivity as well as self-criticism and respect for the families, who entrust themselves to us. We have been able to implement this with children who suffer from eating disorders or tube dependency. As a continuation of the long-standing activity at the Medical University of Graz the spin-off firm Notube.com is presented, which offers treatment for children from 0 to 8 years of age suffering from eating disorders of all kinds. The experience of psychosomatic understanding and treatment acquired at the clinic can thus—even after retirement—continue to benefit hundreds of families. By establishing an outpatient clinic and by offering telemedical support to families this is a medicine that points the way to the future, especially in times of the Covid 19 pandemic.

Lange haben mich die weiten Arme dieser Klinik umfasst. Sie bestimmten mein Leben, mein Denken und mein Handeln. Ich war Teil dieses Hauses, wusste und dachte zwar, dass ich hier ebenso austauschbar wäre, wie alle; aber noch während ich das dachte, da fühlte ich mich schon wieder wichtig. Menschen kamen zu mir, oft von weit her und erzählten, dass sie nirgends so aufgenommen, nirgends so angenommen worden wären. Das alles führte dazu, dass man, bewusst über die Unwichtigkeit seiner selbst und in Kenntnis des Ablaufdatums nur allzu gern bereit war, sich einmalig vorzukommen, schon, weil es im europäischen Individualismus keine Alternative gibt.

Jetzt stehe ich wieder vor Ihnen. Die altvertrauten Bänke, die abgebrochenen Ablagen, der Geruch des Hörsaals in dem einst noch Diapositive gezeigt wurden und Babys zur Demonstration vor den Augen gelangweilter Studiosi demonstriert wurden – er bedrängt mich, macht mich unruhig. Wie in den vielen Jahren, in denen ich nach Wien kam und mich wie in eine Windel zurückversetzt fühlte, die mir noch einmal umgeschnallt wurde – nass und feucht noch von den Ausscheidungen der Jugendzeit, sicher noch mit waschbaren Baumwolltüchern, bestenfalls mit einer Plastikhose umgeben, die gegen das Ausrinnen schützt. So fühlte ich das, wenn mich die Welt oder ich selbst hinderten am Weggehen, am Weiterziehen, am Neubeginn. Die kostbaren Tage verstreichen, die Energie, die langsam entweichen will, wird benutzt und mit jedem verstrichenen Tag, in dem man nichts Neues beginnen durfte, tritt der Tod näher heran und man weiß, dass die Tage gezählt sind, so wie man wusste, dass man hier ausgespuckt werden wird, wie ein Kirschkern.

## Kinderklinik

Dem Haus ergeht es so, wie mir – man weiß mehr, als man umsetzen kann. Keine Kinderklinik benötigt diese Größe. Keine Klinik braucht so viele Betten. Lange schon weiß man, bespricht man, dass Macht und Ansehen nicht von der Anzahl an bespielten Betten abhängt. Lange schon weiß man, dass die Zukunft der Pädiatrie in der lokalen Beratung, im ambulanten Setting und in der Onlineberatung verunsicherter Eltern liegen wird. Man kann in andere Länder schauen, in der das meist schon verwirklicht ist. Dennoch: Die Betten und die Abteilungen ragen wie einst die trotzigen Burgen in die Landschaft. Grafen und höfisches Gesindel ranken sich um sie, das „ancien regime“ lebt und gedeiht ungeachtet der Stürme der neuen Zeit, die an ihre Wälle und Gräben brausen. Das will aber keineswegs heißen, dass die Art der Medizin, die Art der Versorgung in Graz schlecht oder falsch war. Sie war nur eine Art die noch aus einer anderen Zeit stammte, eine stationäre Medizin die sich aus vielen Gründen noch nicht umstellen konnte. Gerade 2020 sieht man wie plötzlich die sogenannten „Überkapazitäten“ zu Vorteilen werden. Jeden als unnötig bezeichnete Bett, jedes intensivmedizinische Bett wird attraktiv und die Einsparungsvorschläge werden plötzlich leise. Allerdings kann die Behandlung der Zukunft auch und vor allem ambulant erfolgen oder telemedizinisch, wenn Hindernisse aus dem Weg geräumt werden, die die Ausbildung der jungen Ärztinnen und Ärzte garantiert und doch dem Spital nur eine Rolle in der Versorgung von Kindern und ihren Familie zuerkennt und nicht diese zentrale, wie ich sie erlebt habe.

## Bleiben wir beim Titel …

Vergessen wir den Titel nicht, sonst redete ich noch über die Bewältigung der Flüchtlingskrise und die allgemein sich verrohende Sprache und Gebärde, das kälter werdende Herz, das sich über in kalten Lachen gewaschene Neugeborene ebenso hinwegsetzen kann, wie über ein Elend an einer verlassenen Grenze und einem Kleinststaat, der das dreckige Geschäft der hinter ihm seienden Reichen besorgt, selbst noch zitternd vor Illegitimität, eine Geschichte zusammenzimmernd, die es so nie gegeben hat, eine nationale Identität, die sich bei Österreich so gar nicht einstellen will und die dort Operettencharakter hätte, wäre da nicht die alltägliche Brutalität von in Phantasieuniformen gesteckter Soldaten, die auf Frauen, Männer und Kinder eindreschen, die in der Nacht über unwegsame Flüsschen und eiskalte Wälder versuchen, das zu erreichen, in dem wir leben – das Paradies des Konsums, der leuchtenden Auslagenscheiben und des überall vorhandenen Internets.

## Wir waren nie besser und werden es nie sein

Mehr als Wasser und Wärme sucht der Flüchtling ein WLAN, einen Anschluss an das Netz, aus dem er Informationen über seine Angehörigen schöpft, Informationen zu neuen Fluchtwegen, irisierende Nachrichten zu Helfern und deren Komplizen bekommt und sich neu einrichtet, ungeachtet dessen, dass schon wieder ein Weg von gutgekleideten, satten Ministern verstellt wurde, ungeachtet dessen, dass die, die gerade noch unter der Knute einer sowjetischen Hegemonie stöhnten, sich nun das Recht der Freien herausnehmen, nämlich herzlos sein zu können und zu dürfen, das, was sie mit Freiheit assoziieren, nämlich ebenso zu sein, wie der Mensch gemacht und gebaut ist und was er dann unmenschlich nennt, obwohl es doch so menschlich ist, dass man es human nennen müsste und nicht inhuman, weil man ansonsten von einem Menschenbild ausginge, das sich selbst der Erfinder des Humanismus Homer nicht vorstellte, denn auch dort sehen wir Menschen und Götter, die vor allem essen, lieben und töten. Wir waren nie besser und werden es nie sein, mag sein, dass Konrad Lorenz ebenso Recht hatte, wie noch mehr Arthur Köstler, die beide einen Fehler in der Maschine Mensch annahmen. Zum Töten und zur Vermehrung ungeachtet der Anzahl der vorhandenen Kopien verurteilt, kriecht dieses Gewürm, dessen Teil ich bin, auf diesem Planeten, zerstört denselben, wissend, dass sie ohne ihn nicht sein können, und selbst in ihren Träumen, die sie sich in Filmen vorspielen, morden sie und brandschatzen und wenn sie dann ruhiger werden, schauen sie sich Kriminalfilme an, die mindestens einen Mord enthalten, besser zwei, weil sonst die Auflösung zu früh kommt und der Traum, das Märchen endet, bevor die Mordlust des Zusehers gestillt ist.

## Anständige Medizin

Doch jetzt: anständige Medizin. Was machen wir, wenn wir kranke Kinder und deren Eltern treffen? Im besten Fall nehmen wir den Kindern die Eltern weg, im noch besseren Fall ihre vertraute Umgebung und legen sie in kalte Betten in schlecht durchlüftete Räume und geben ihnen allerlei. Infusionen, Überwachungsgeräte und Erfolge machen uns besser als das, was die Eltern anzubieten haben. Wir sind die wahren Retter der Kinder, und die Eltern sind es nur, insoweit sie unseren Ratschlägen folgen, unsere Beratung annehmen und so ihren Kindern die besten Chancen geben. Das ist komisch. Wir sind nur so mächtig geworden, weil das Wissen um Richtig und Falsch verloren gegangen ist. „Der Verlust der Mitte“ (1948) dieses wenig bekannte und doch so lesenswerte Buch des österreichischen Kulturphilosophen Hans Sedlmayer ist nun von einer befürchteten Zukunft Realität geworden. Eltern wissen nicht mehr, was sie machen sollen. Sie halten sich an neuen Versatzstücken fest, die wir ihnen liefern. Kindergesundheit, Verhinderung von Kinderunfällen, Kindersitze in Autos, langsames Zusehen der kindlichen Entwicklung und anderes mehr.

Alter Wein in neuen Schläuchen wird angeboten, J. J. Rousseau (1712–1778), der Erzieher der französischen Revolution, der Erfinder der Erziehung eines Kindes, von dem er annahm, das es alle Wuchskompetenz schon mit sich brächte, kommt immer wieder. Immer wieder wird Erziehung und Betreuung des Kindes als ein Freilegen der im Kinde innewohnenden Kräfte angesehen, als ein Wegschaufeln des gesellschaftlichen Detritus, der sich über das Kind legt. So verstehen wir die Diskussionen über die Nutzung des Internets und den Gebrauch der sozialen Medien, die Eltern mit uns Kinder- und Jugendärzten führen; so verstehen wir die Unsicherheit der Eltern, die keine Vormütter und Tanten mehr haben, denen sie vertrauen und deren Betreuungskonzepte sie annehmen können.

## Früh- und Mangelgeborene werden gerettet

Und dann retten wir Kinder, die es noch vor 20 Jahren nicht gegeben hätte. Früh- und Mangelgeborene, Kinder, die in der 23. Woche zur Welt kommen müssen und die weniger als 500 g wiegen. Kinder, die ein Herz haben, das nur eine Kammer hat, oder die Syndrome haben, die noch wenig beschrieben sind, inzwischen gut verstanden, und die nichts hören, manchmal nichts sehen und die dann doch geliebt werden – einfach, weil der Mensch nicht anders kann, als seine Brut zu lieben, sei sie auch missraten oder zumindest schwach. Keiner, der sich mehr zum pater familias aufschwingt, der nach der Geburt über Leben oder Tod des Neugeborenen entscheidet, keiner, der sich der ethischen Herausforderung stellen könnte, das zu unterlassen, was möglich ist; keiner, der wüsste, was aus dem einen oder anderen Kind werden wird, und der sich daher in die Position begeben könnte, sich, den Familien und der Menschheit etwas zu ersparen, das er als Sieg sieht, sehen muss und sehen will.

## Beatmet, operiert, wieder operiert …

Nicht nur im Schweiße des Angesichts wurden diese Kinder geboren unter Schmerzen, wie die Verwünschungen des Allerhöchsten in der hebräischen Bibel heißen, nein, sie wurden im Schweiße der vielen Assistenten, Oberärzte und Professoren und mit den Erfindungen der Neuzeit zum Leben gezwungen, das sie nun besser oder schlechter führen. Das sind die Kinder, die wir kennenlernen. Beatmet, operiert, wieder operiert, mit künstlicher Ernährung, die ihnen durch die Nase oder durch die Bauchwand angeboten wird, gut berechnet, die Physiologie der Muttermilch nachahmend, dann gesund voller Vitamine und Spurenelemente, angereichert, oder nur die einzelnen Aminosäuren, die direkt resorbierbar sind, oder spezielle Darreichungen von Fetten in kurzen oder langen Ketten, alles zum Besten des Kindes.

## Ziele und Richtlinien

Leider reagieren nicht alle Kinder gut darauf: weitere Ziele und Richtlinien, an die wir uns halten sollen oder glauben zu müssen, hindern uns Ärztinnen und Ärzte, auf das Kind einzugehen: Perzentilen und Wachstumskurven werden zu Diktatoren, die uns übersehen lassen, dass das Kind sich erbricht, sich durchstreckt, weil es schon übervoll ist und ihm die Nahrung in die Speiseröhre zurückrinnt, was große Schmerzen zur Folge hat, und statt ihm weniger zu geben oder ihn essen zu lehren, geben wir Protonenpumpenhemmer, die zu mehr intestinalen Allergien führen, aber doch die Magensäure reduzieren, auch und gerade wenn diese dringend benötigt würde, aber zurückrinnt einfach, weil wir zu viel geben. Wir machen das aber nicht, weil wir den Kindern schaden wollen, sondern in bester Absicht und denken dann auch nicht an Hannah Arendt, die uns zu zeigen versucht hat, das nur der wahrhaft gefährlich wird, der dies in bester Absicht macht, denn der Verbrecher weiß um die Schändlichkeit seines Tuns und wird entweder erwischt oder lässt es – einfach, weil er weiß, dass es Unrecht ist. So geht es uns nicht. Wir sind im Wissen um unsere Berechtigung und unsere gesellschaftliche Stellung uneinnehmbar und vor der Mächtigkeit der Realität, vor der Unerbittlichkeit des kalten Gangs der Realität (Robert Schindel), vor dieser füttern wir unerbittlich.

## Kinder essen lehren

Da kommen sie denn daher von überall und durch die unerklärlichen Gewinde des Netzes zu uns – die Eltern, die nicht wissen, wie sie es machen sollen, wie sie ihrem Kind, das doch so speziell und einmalig ist, *essen lehren* sollen, die schon überall waren: in Maulbronn bei den Psychiatern, in Kliniken und Praxen von Logopädinnen und Ergotherapeutinnen, von Alternativmedizinern und Elternberatern und alle konnten sich nicht hinwegsetzen über die Erfordernisse der Ärzte, über deren Wahrspruch und deren Kurven und Ziele und der Versprechung einer gesunden Zukunft, eines schönen, gerade gewachsenen Kindes und einer glücklichen Familie, wie immer die auch aussehen mag.

Dann geriet die Beratung zu einer Überredung, einem Wort, in dem schon die Unterredung enthalten ist, also einem Machtverhältnis, in dem wir oben sind und die unten und sich am besten an unsere Ratschläge halten. So ist der übliche Beratungskontext verständlich: da ein Oben, dort ein Unten; da eine Frage, dort ein Schlag und sei es nur ein Rat-schlag.

## „Parents empowerment“

Das scheinbar neue Wort hieß dann: „parents empowerment“, was nichts anderes hieß, als dass wir den Eltern wieder zutrauen, ihre Kinder zu verstehen, in ihnen zu lesen und ihre Wünsche und Bedürfnisse zu dekodieren. Sicher, das ist schwer, besonders, wenn wir ihnen die Kinder weggenommen hatten, aber das wissen wir nun schon und wissen auch, dass wir das so nicht machen hätten dürfen, es aber nicht besser verstanden haben.

## NoTube

Im Grunde machen wir bei NoTube das, was wir auch in diesem Haus gemacht haben: Wir kennen die Medizin und ihre wunderbaren Kenntnisse und ihre Grenzen. Wir kennen die Eltern und haben ihnen immer zugehört. Wir freuen uns an den Babys und Kleinkindern, an den Schulkindern und den Jugendlichen und nehmen sie ernst, so weit, wie man jeden Menschen ernst nehmen kann. Wir glauben, dass der Mensch in seinem dunklen Drange sich des rechten Weges wohl bewusst ist und fallen auf die Worte J. W. Goethes aus Faust herein, die dieser dem Herrn selbst in den Mund legt. Genau das machen wir, online, in einem *Learn-to-Eat*-Programm und seit zwei Jahren auch in *Esslernschulen*, die ein Ambulatorium und eine Schule sind, aber ohne eines von beiden ganz zu sein.

Verunsichert, verloren, ängstlich kommen die Eltern zu uns. Aktenordner voller Befunde tragen sie mit sich. Man hat ihnen Aufgaben übergeben, nachdem man heldenhaft und oft ungefragt ein Leben lebbar, manchmal sogar lebenswert gemacht hat, das ohne uns Ärzten nie stattgefunden hätte. Jetzt wollen sie Antworten. Sie fragen uns: „Was ist das Beste für unser Kind?“, „Wieviel muss ich geben?“, „Wird das Kind ausreichend hydriert?“, „Was mache ich, wenn es erbricht?“, „Muss ich die Flüssigkeit ersetzen?“ Das sind nur einige der Fragen und was sollen wir machen? Wir wissen die meisten Antworten nicht, wie auch unsere Kolleginnen und Kollegen sie nicht wussten. Die aber, die taten so, als ob sie es wüssten. Wir stehen dann als die Dummen da, die zu wenig bieten, zu wenig wissen und kennen. Doch die vielen Titel, die wir angehäuft haben, die Publikationen in den Fachjournalen, die uns ausweisen, die Anerkennungen weltweit und das Gerücht, das wir das können – sie alle verhindern, dass man uns andauernd nach Bestätigungen und Nachweisen fragt, die so nicht erbracht werden können.

## Elternberatung bei Ernährungsstörungen

Die Gegenwart der Elternberatung bei Essstörungen im Kleinkindesalter ist, dass es welche gibt, die wissen, und welche, die nicht wissen, und daher zu folgen haben.

In alten Zeiten wurde das Wissensgefälle einfach aufrechterhalten: Der jüdische Kalender zum Beispiel ist so kompliziert, das ihn nur Wenige verstehen und berechnen können. Da sich aber nach dem Kalender alle Jahresfeste, Todestage und anderes Bedeutsames richten, war die Bekanntgabe der Termine auf die Versammlung der Gelehrten in Jerusalem oder Safed beschränkt, auf die die Gemeinden allerorten referenzierten und sich beriefen. Genau so ergeht es heute: Wird ein Beitrag in einem hochwertigen Journal veröffentlicht, ist der Annahme, die meist auf Wahrscheinlichkeitsrechnungen beruht, Wahrheit gegeben worden. Aus der Konstruktion einer Wirklichkeit ist nun eine Wirklichkeitskonstruktion geworden und indem sie vorhanden ist, bestimmt sie die Wahrnehmung der Wirklichkeit und die Reaktion darauf.

Das könnte man „depowerment“ nennen; sowohl die Ärztinnen und Ärzte als auch die Eltern haben diesen Annahmen, die nun Wirklichkeiten sind, zu folgen, sie sind zu Leitlinien geworden. Wie I. D. Mutz sagt: *„Nur, wo wir wenig, oder nichts wissen, haben wir Leitlinien. Wo wir wissen, brauchen wir keine!“* So einfach ist das. Keiner weiß, ob ein überfüttertes und erbrechendes Kind klüger wird. Keiner weiß, ob es das *„catch up growth“* bei Kindern gibt, die bereits in der ersten Hälfte der Schwangerschaft zu klein waren und dann mittels Kaiserschnitt als „small for date“ geboren wurden; keiner weiß, ob die Wahrscheinlichkeitsrechnung stimmt und selbst wenn sie stimmen sollte, ob sie auf dieses Individuum anwendbar wäre; und selbst wenn sie anwendbar wäre, ob es dem Kind hülfe; und selbst wenn es dem Kind hülfe, ob es dann ein glücklicherer oder sogar besserer Mensch werden würde. Alles das ist unbekannt. Nichtsdestotrotz halten wir uns an das, weil auch wir unsicher geworden sind, ängstlich und leitlinienverliebt.

## Die Zukunft der Elternberatung

Ausgeschieden aus den heiligen Hallen der gelebten Wissenschaft behaupte ich, so wie ich es vertrat und dachte, selbst als ich noch Teil war: Die Zukunft der Elternberatung wird die fast unhörbaren und unwahrnehmbaren Fähigkeiten der Eltern wieder annehmen müssen, ihre Feinfühligkeit und ihr Verständnis für ihr Kind respektieren müssen. Sie wird – und das ist der Grund, warum in diesem Haus keine Kinder mit Sonden nach Hause gehen – den Eltern ihr Kind zurückgeben müssen, sie in ihrer Zeitlichkeit und ihrem Selbstverständnis, ihrer Lebensbewältigung und ihrem Charme respektieren müssen. Sie wird uns zurücklassen als Hebammen der Erziehung, meutisch, wie es Sokrates vorschlug, fragend, zweifelnd, unsicher den Weg eines Kindes begleitend, das wir so nicht verstehen können, das sich anders ernährt, anders lebt und anders denkt, als wir es uns vorstellen können. Wir werden diesen Weg mit allen Mitteln gehen müssen: ambulant in Ordinationen niedergelassener Kinderärztinnen und -ärzte, in Kliniken und Spitälern und telemedizinisch. Diese letzte Variante wurde von uns seit 2010 angeboten als es noch praeter legem war. Marguerite Dunitz-Scheer begann mit der Hilfe eines datenaffinen Studenten ein Internetservice anzubieten, das viele E‑Mail-Kontakte ersetzte. Langsam wurde die webpage besser, mehr und mehr Familien vernetzten sich, datenmäßige Mundpropaganda griff um sich. Wir besuchten viele Länder in und außerhalb Europas, hielten Vorträge, berieten Institutionen wie sie mit essgestörten Kindern umgehen sollten. Wir publizierten erstmals den Begiff: „Sondendependenz“ als unerwünschte Folgewirkung einer enteralen Ernährung und er wurde in den Kanon der Medizin aufgenommen. Gerade die telemedizinische Betreuung benötig Feingefühl für die Bedeutung von Worten, ein Lesen zwischen den Zeilen. Die Ergänzung mittels Videoaufzeichnungen von Essen und Spielen gaben den Kindern den Raum in dem sie erkenn- und behandelbar wurden. Der Arzt im Wohnzimmer des Kindes, der dessen Wachen, Spielen und Essen beobachtet, allenfalls beeinflusst und im elektronischen Austauch mit den Eltern respektvoll deren Umgang, aber auch die Ängste und die Verzweiflung anspricht, bearbeitet, lindert und löst. Dazu bedarf es eines Teams, Mitarbeiterinnen die diesen Weg mitgegangen sind und weiterhin mitgehen. 2020 wurde die „Firma“ 10 Jahre alt. Etwa 90 Kinder werden im tagesklinisch geführten Ambulatorium pro Jahr behandelt über 110 telemedizinisch. Entscheidend ist, dass in beiden Settings derselbe Zugang zum Patienten, zu den Familien eingehalten wird: sie sind im Recht und wir geben Anregungen, machen Vorschläge, bearbeiten Hindernisse und haben daselbe Ziel wie die Eltern: ein so weit als möglich glückliches Kind.

Machen wir das alles, so wird die Morgenröte der Zusammenarbeit auf ähnlicher Augenhöhe mit den Eltern der Kinder erscheinen, Aurora des tiefen Wissens um die Notwendigkeit der starken Elternschaft und wir werden uns scheu zurückziehen auf das Wenige, das wir zu wissen vermeinen. Wir werden ehrfürchtig vor den Aufgaben stehen, die wir diesen Eltern aufgegeben haben und beschließen sie nicht weiter zu quälen mit unseren Anerkennungswünschen für die Tage und Nächte, da wir ihr Kind ins Leben begleitet, manchmal es zum Leben gezwungen haben.

Ich möchte meinen Vortrag und letzten Endes auch diese kleine Tagung (anlässlich meiner Versetzung in den Ruhestand) mit dem Motto dieser Universität in Graz schließen: *„Salus aegroti suprema lex“.*

